# Incidence of thromboembolism in children with primary nephrotic syndrome: a systematic review and metaanalysis

**DOI:** 10.1186/s12882-023-03160-y

**Published:** 2023-05-01

**Authors:** Zhen Wang, Han-Yun Tang, Qiang Lin, Xiao-Zhong Li

**Affiliations:** 1 Department of Renal Immunology, Children’s Hospital of Soochow University, Soochow, JiangSu China; 2 Department of Pediatrics, Zibo Maternal and Child Health Hospital, Zibo ShanDong, China

**Keywords:** Primary nephrotic syndrome, Children, Thromboembolism, Prevalence

## Abstract

**Objective:**

To estimate the incidence of thromboembolism in children with primary nephrotic syndrome with Meta-analysis.

**Methods:**

Relevant studies published from January 1, 1980 to December 31, 2021 were retrieved from Pubmed, Web of science, Cochrane library, China National Knowledge Infrastructure (CNKI), China Science and Technology Journal Database(VIP) and Wangfang Database. Quality evaluation of the literatures included was conducted according to Agency for Healthcare Research and Quality(AHRQ) assessment tool, followed by data extraction and Meta-analysis with software RevMan 5.3.

**Results:**

A total of seven studies involving 3675 subjects were included. The overall prevalence was 4.9% with 95% CI of 2.83 to 7.05.However, a significant heterogeneity (P < 0.001) was observed with *I*^2^ = 89%. The prevalence of venous thromboembolism was 3.3% with 95% CI of 1.7 to 4.9. The prevalence of arterial thromboembolism was 0.5% with 95% CI of 0.2 to 1.4.

**Conclusion:**

Children with nephrotic syndrome are prone to thromboembolism, and it may lead to disability or death, therefore prevention measures is critical to decreasing the prevalence of thromboembolism.

**Supplementary Information:**

The online version contains supplementary material available at 10.1186/s12882-023-03160-y.

## Background

Primary nephrotic syndrome (PNS) describes a group of clinical syndromes presenting with massive proteinuria accompanied by edema, hypoalbuminemia, and hyperlipidemia. Thromboembolism (TE) is one of the major complications of PNS, which is considered a serious life-threatening complication of NS in addition to infection. Although its prevalence is not as high as infection, the higher disability and mortality related to TE attracted the attention of clinicians [1–6]. It was reported that the mortality of NS with TE in children is approximate 8.5% [7], and especially for cerebral venous thrombosis, the mortality is as high as 10% [8]; therefore early management is crucial. The prevalence of venous thromboembolism (VTE) is significantly higher than that of arterial thromboembolism (ATE) in pediatric patients, the common types of which include renal venous thrombosis (RVT), deep venous thrombosis (DVT), pulmonary embolism (PE), and cerebral venous thrombosis (CVT). It has been reported that the prevalence of TE in pediatric patients with NS is 1.8%¬-5% [6, 9, 10]; however, relevant reports are few and varied. Observational studies on this issue are rare worldwide, and even fewer systematic reviews have been carried out. Currently, there is only one report on TE in children with PNS infected with the severe acute respiratory syndrome coronavirus 2 (SARS‑CoV‑2, the causative agent of COVID-19) [11], and TE in patients with PNS after COVID-19 vaccination has only been reported in adults [12]. However, as risk factors for TE, infection with SARS‑CoV‑2 or vaccination with a COVID-19 vaccine might increase the occurrence of TE in children with PNS. Therefore, in the current stage of widespread transmission of SARS‑CoV‑2, TE in children with PNS should receive more attention from clinicians.

The goal of this paper is to provide a comprehensive review of the current literature in terms of TE prevalence in children with PNS, to allow clinicians to carry the optimum management to reduce the prevalence of TE.

## Methods

### Search strategy and study selection

We performed a comprehensive search of the Pubmed, Cochrane Library, Web of science, CNKI, VIP, and Wanfang datebases to identify relevant studies from 1 to 1980 until 31 November 2021 using the following search term: ((Nephrotic Syndrome) OR (Nephrotic Syndromes) OR (Syndrome, Nephrotic) OR (Syndromes, Nephrotic)) AND (Thromboembolic OR Thromboembolism OR thromboembolisms OR thrombosis) AND (child or children or childhood). Inclusion criteria included: Patients under 21 years old; with a diagnosis of PNS; and thromboembolism diagnosed definitively by imaging examination. Exclusion criteria included: Case reports or review articles; patients with secondary NS; Data duplication; and articles without available full texts. Two independent reviewers scanned the titles and abstracts against our inclusion and exclusion criteria to select potential articles. Full texts of the eligible articles were then retrieved and reviewed for final inclusion. Disagreements between the two reviewers were resolved after consultation with the third investigator.

### Data extraction

Data were extracted from the included studies using an excel sheet, and included the article title, the first author’s name, the year of the publication, sex, area, sample size, incidence of thromboembolism.

### Quality assessment

Two independent reviewers evaluated the risk of bias in the included studies using the Agency for Healthcare Research and Quality (AHRQ) assessment tool [13]. Quality assessment of cross-sectional studies was obtained through a scoring system including 11 questions. Articles scoring 8 to 11 were considered as high-quality articles, articles scoring below 4 were considered as low quality articles and the rest were considered fair [14].

### Statistical analysis

Review Manager (RevMan)[Computer program. Version 5.3, The Cochrane Collaboration, 2014] was used to conduct the meta-analyses. The *I*^2^ test was used to evaluate the heterogeneity of the selected studies. Heterogeneity was divided into three classes of less than 25% (low heterogeneity), 25–75% (moderate heterogeneity), and more than 75% (high heterogeneity). Funnel plots were used to identify publication bias.

## Results

### Search results

**Fig. 1:**
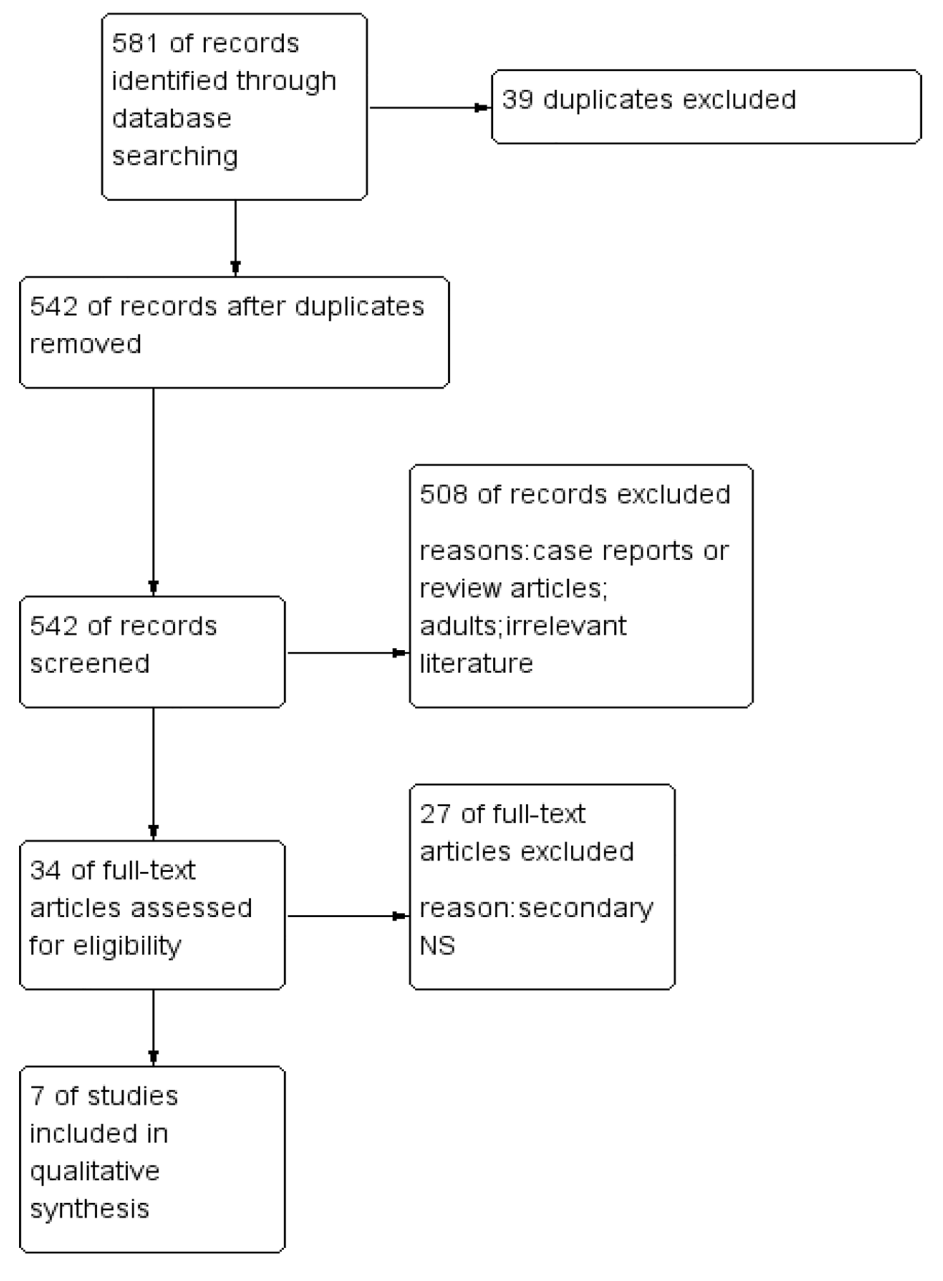
Flow diagram of the search and review process

Using the search strategy mentioned in the methods section, we obtained 581 records from the 6 databases. After removal of 39 duplicates via endnote software, 542 records were obtained. Title and abstract screening resulted in the inclusion of 34 and the exclusion of 508 reports. Then, 27 studies were excluded after the full-text articles were assessed for eligibility. In total, seven studies [15–21] were eligible for quantitative synthesis (Fig. 1).

### Quality assessment and characteristics of the included studies

There were six retrospective cohort studies and 1 case series study. The specifications of the selected articles are presented in Tables 1 and 2.

**Table 1:** Characteristics of the included studies

Author, year	region	event(male)	Sample size	Male/Female	AHRQ
Mehls,1987	Germany	9(5)	204	121/83	7
Marusia,2000	Bulgaria	9(4)	436	NR	7
Bryce A,2009	US	16(7)	244	168/158	9
Betul,2015	Turkey	17(16)	188	108/80	8
Liu,2016	China	32(24)	238	NR	7
Shannon,2019	North America	11(5)	370	227/143	8
Lv,2020	China	27(21)	1995	NR	8

NR = not reported

**Table 2:** Events of each type of thrombosis

Author,year		Events of main types of thrombosis						Total cases	Death
	RVT		DVT	PE	CVT	VTE	ATE		
Mehls,1987	1		3	2	0	6	5	9	NR
Marusia,2000	0		9	0	0	9	1	9	2
Bryce A,2009	0		12	4	0	16	1	16	NR
Betul,2015	NR		5	NR	4	15	2	17	2
Liu,2016	2		12	3	NR	NR	NR	32	1
Shannon,2019	1		NR	1	3	11	NR	11	NR
Lv,2020	9		6	7	6	24	3	27	0

NR = not reported;some have multiple thromboembolisms

### Meta-analysis(incidence of TE in children with PNS)

**Fig. 2:**
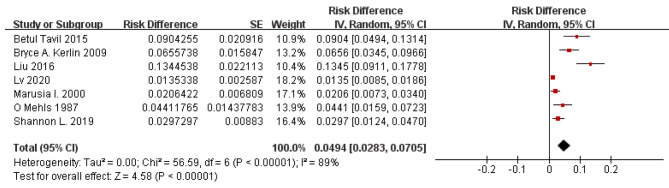
Forest plot of TE incidence in children with PNS

According to the results of the study assessed using a forest plot, the overall incidence of TE in children with PNS was 4.9% with a 95% confidence interval (CI) of 2.83 to 7.05. Significant heterogeneity (*P* < 0.001) was observed with *I*^2^ = 89% (Fig. 2).

### Sub-group analysis

Subgroup analysis was performed for different characteristics, such as region, sex, thrombosis type, and year of publication. The results showed that the incidence of TE was significantly different between regions, thrombosis types, and years of publication. However, no difference was observed in the incidence between males and females (Table 3). The heterogeneity of the above subgroup analysis was still large, indicating that region, sex, thrombosis type, and year of publication were not the main reasons for the heterogeneity. Due to lack of detailed data, further sub-group analysis could not be carried out. Factors such as the duration after the onset of nephrotic syndrome, status of proteinuria, and use of medication may also influence the incidence of thromboembolism. And above factors may partly explain the significant heterogeneity. Therefore, the reported incidence in children with PNS may be over- or under-estimated due to potential selection bias. Thus, the reasons for the observed heterogeneity were complex and require further study.

**Table 3:** Sub-group analysis of the incidence of thromboembolism in children with PNS

Sub-group	Items	Studies included		Heterogeneity		Incidence %(*CI*)
				*I*^2^(%)	*P* value	
Region	China	2	95		<0.01	7.2%(-0.046, 0.19)
	Abroad	5	75		<0.01	4.6%(0.024, 0.065)
Gender	Male	5	96		<0.01	5.18%(0.0234, 0.1146)
Thrombosis type	Female VTE ATE	5 6 5	98 81 100		<0.01 <0.01 <0.01	3.63%(0.0193, 0.682) 3.28%(0.0172, 0.0483) 0.52%(0.002, 0.0136)
Year of publication	Before 2017	5	89		<0.01	6.76%(0.03, 0.1053)
	After 2017	2	68		<0.01	1.94%(0.0042, 0.0347)

### Sensitivity analysis and publication bias

**Fig. 3:**
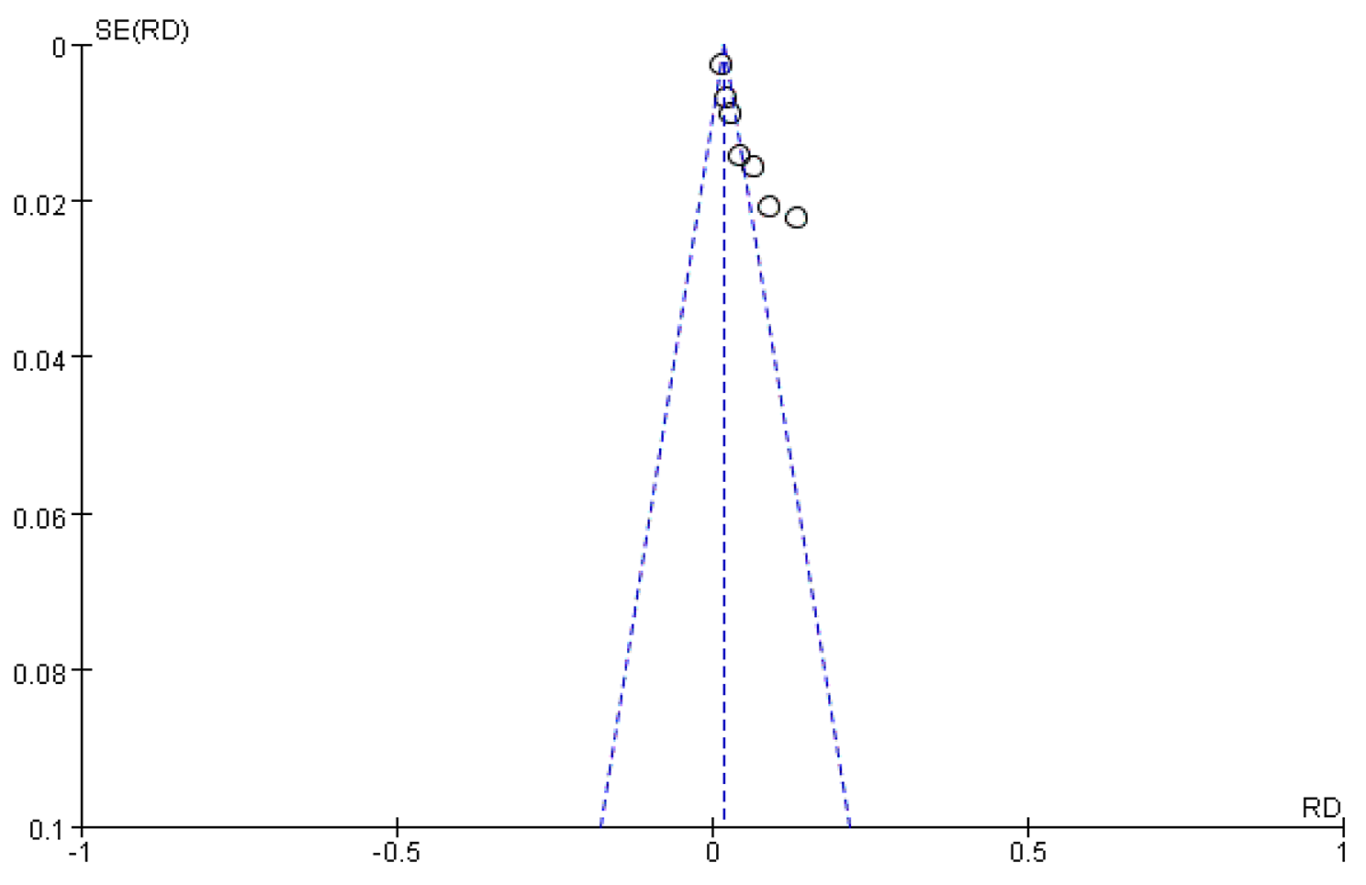
Funnel plot of the results of the incidence of TE in children with PNS

Sensitivity analysis was conducted using one-by-one exclusion. Excluding one study at any time had little impact on the pooled effect value and *I*^2^ based on the random effect model, and the conclusions were not significantly changed, indicating that the meta-analysis results of this study were relatively stable. Only seven studies were included; therefore, we did not carry out Egger’s regression test. However, we still created a funnel plot using RevMan 5.3, which showed a risk of bias (Fig. 3).

### Meta-analysis(incidence of VTE in children with PNS)

**Fig. 4:**
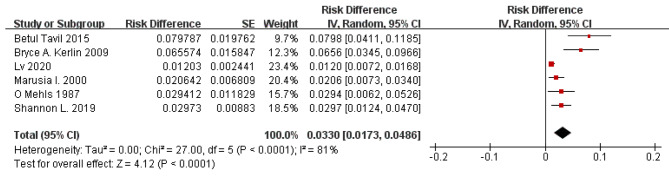
Forest plot of VTE incidence in children with PNS

According to the results of the study shown in the forest plot, the overall incidence of VTE in children with PNS was 3.3% with a 95% CI of 1.73 to 4.86. Significant heterogeneity (P < 0.001) was observed with *I*^2^ = 81% (Fig. 4).

### Meta-analysis (incidence of ATE in children with PNS)

**Fig. 5:**
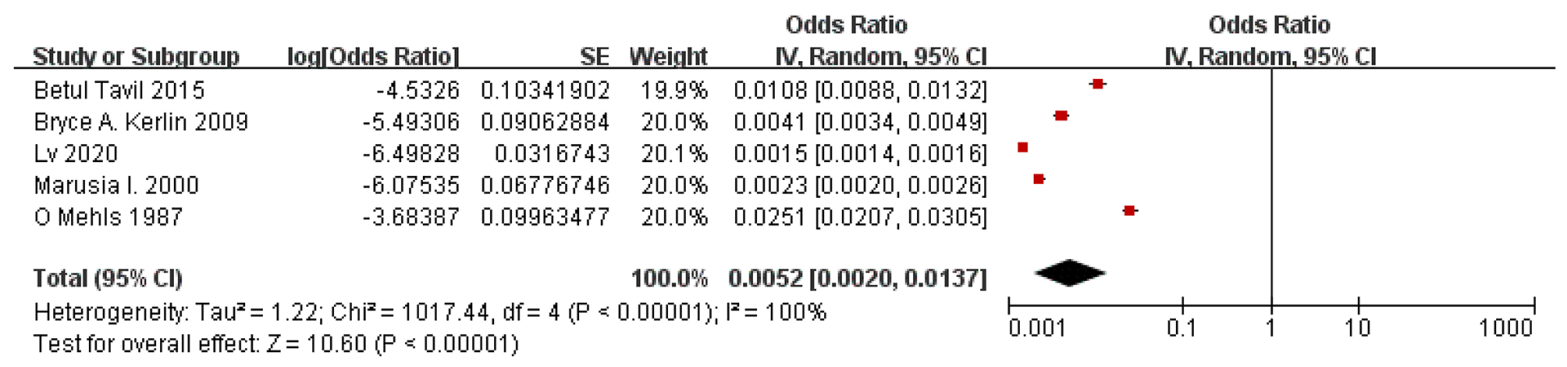
Forest plot of ATE incidence in children with PNS

According to the results of the study shown in the in forest plot, the overall incidence of ATE in children with PNS was 0.52% with a 95% CI of 0.2 to 1.37. Significant heterogeneity (P < 0.001) was observed with *I*^2^ = 100%.(Fig. 5).

### Meta-analysis(incidence of DVT in children with PNS)

**Fig. 6:**
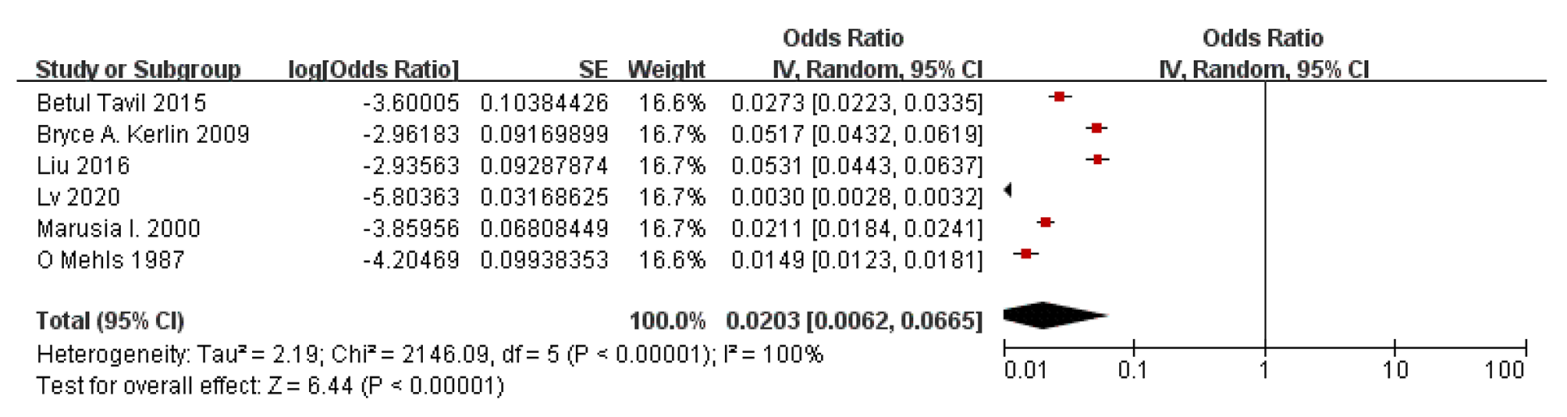
Forest plot of DVT incidence in children with PNS

According to the results of the study in forest plot, the overall incidence of ATE in children with PNS was 2.03% with 95% CI of 0.62 to 6.65. A significant heterogeneity (*P* < 0.001) was observed with *I*^2^ = 100% (Fig. 6).

### Meta-analysis(incidence of PE in children with PNS)

**Fig. 7:**
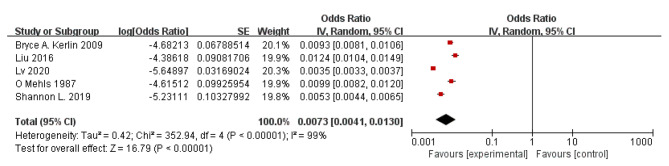
Forest plot of PE incidence in children with PNS

According to the results of the study shown in the forest plot, the overall incidence of ATE in children with PNS was 0.73% with a 95% CI of 0.41 to 1.3. Significant heterogeneity (P < 0.001) was observed with *I*^2^ = 99% (Fig. 7).

## Discussion

There are many case reports of children with PNS complicated with TE; however, few large observational studies have been conducted. Although the incidence of PNS with TE is low in children, many deaths associated with it have been reported. A total of 121 cases of TE occurred in the literature included in this study, among which 5 deaths were reported [16, 20, 21], and some cases had thrombus related sequelae.

In this study, the overall incidence of TE in PNS in children worldwide was 4.9%, which was close to the incidence reported in previous studies [6, 9, 10]. The studies conducted by Betul et al. [16] and Liu et al. [21] reported a significantly higher incidence of thromboembolism (9% and 13%), which might have been caused by the pathological type of nephropathy of the included cases [22, 23]. Studies suggest that the incidence in males is higher: The study conducted by Betul et al. [16] showed significant differences between males and females, which might have been caused by admission bias. In our study, the incidence of TE in children with PNS was observed to be higher in China than in other countries. However, only two Chinese studies were included in this analysis, and the incidences in the two studies were quite different; therefore, the results should be interpreted prudently. In the present meta-analysis, the incidence reported by literature published before 2017 was higher than that published after 2017. However, through careful analysis of the studies, we found that the year of publication was inconsistent with the age of the children included in the study. For example, the study conducted by Lv et al. [19] included cases from 1993 to 2019, so the results could not represent a decline in the incidence of TE. In addition, the incidence of VTE was found to be 3.3%, which was close to that of previous reports [15, 24] and higher than that of arterial embolism. This might have been caused by the slower flow in the vein, the presence of venous valves, the thin wall of the vein, and the high viscosity of the blood in the vein. Our study suggested that the incidence of DVT in children with PNS was 2.03%, which was lower than the reported incidence of 7% [25]. This was considered to related to the exclusion of cases with secondary nephropathy. The incidence of PE in children with PNS is 0.73%. Although the incidence of PE was lower, its associated mortality was higher [26].

Several factors have been identified to be associated with the development of thromboembolic disease in NS. Multivariate analysis shows that age ≥ 12 years and severe proteinuria are independent risk factors for thromboembolism [17]. Membranous nephropathy is the most common pathological type complicated by thromboembolism.Moreover, genetic predisposition may not be related to the occurrence of nephrotic syndrome in children, but it plays a significant role on thromboembolism after NS [27]. Secondary NS is more prone to thrombosis than primary NS.

There are still many controversies on whether NS patients need prophylactic anticoagulant drugs, the timing of administration, drug selection and drug dosage. The latest guidelines issued by 2021 KDIGO clearly put forward the algorithm of anticoagulant prophylaxis for MN patients. In the guidelines, it is proposed that the timing and drug selection of medication should be determined according to serum protein concentration, risk factors of thrombosis and bleeding risk [28]. No recommendations are made for other pathological types of NS. Evidence supporting recommendations for pediatric antithrombotic therapy is still weak. In children with VTE, the American College of Chest Physicians guidelines suggests that thrombolysis therapy be used only for life- or limb-threatening thrombosis [29].

Our study had several limitations: (i) The number of studies included was small and the sample size was not large enough. Significant heterogeneity was found that could not be explained by the differences in region, sex, thrombosis type, and year of publication; (ii) Data in some of the included studies were not complete;iii) Image investigation was typically performed in symptomatic patients, and not universally performed in all NS patients, which may underestimate the true incidence of thromboembolic complications in children with PNS.

## Conclusion

The results of this study showed that the incidence of TE in PNS in children was 4.9%. To improve the quality of life and the prognosis of children with PNS, more attention should be paid to the incidence of TE. In addition, more studies should be conducted on risk factors, pathophysiology, and measures to prevent and treat TE, with the aim of reducing the incidence of TE.

## Electronic supplementary material

Below is the link to the electronic supplementary material.

Supplementary Material 1

## Data Availability

The datasets generated and/or analysed during the current study are available from corresponding author on reasonable request.

## Abbreviations

TE: Thromboembolism
PNS: Primary nephrotic syndrome
VTE: Venous thromboembolism
ATE: Arterial thromboembolism
RVT: Renal venous thrombosis
DVT: Deep venous thrombosis
PE: Pulmonary embolism
CVT: cerebral venous thrombosis
RevMan: Review Manager
CNKI: China National Knowledge Infrastructure
VIP: China Science and Technology Journal Database

## References

[1] Xin-Hua W, Hai-Juan H, Lin-Mei ZH. Clinical analysis of twelve cases with cerebral venous sinus thrombosis in children.Chin J Neurol.2013;46(6):383–386.doi:10.3760/cma.j.issn.1006-7876. 2013. 06.007.

[2] Guenther RA, Kemp WL (2018). Delayed death due to Saddle Pulmonary Thromboembolus in Child with Nephrotic Syndrome Induced by Focal Segmental Glomerulosclerosis. Am J Forensic Med Pathol.

[3] Wright JM, Watts RG (2011). Venous thromboembolism in pediatric patients: epidemiologic data from a pediatric tertiary care center in Alabama. J Pediatr Hematol Oncol.

[4] Torres RA, Torres BR, de Castilho AS (2014). Venous sinus thrombosis in a child with nephrotic syndrome: a case report and literature review. Rev Bras Ter Intensiva.

[5] Boussetta A, Jaber C, Jellouli M, Gargah T. Thromboembolic complications in children with primary nephrotic syndrome: A Tunisian series. Tunis Med. 2022 Janvier;100(1):33–36. PMC8996312 35822329

[6] Han KH, Park JY, Min SK. Bilateral iliac and popliteal arterial thrombosis in a child with focal segmental glomerulosclerosis. Korean J Pediatr. 2016;59(5):242-5. doi:10.3345/kjp. 2016.59. 5.242. 10.3345/kjp.2016.59.5.242 PMC4897161 27279890

[7] Suri D, Ahluwalia J, Saxena AK (2014). Thromboembolic complications in childhood nephrotic syndrome: a clinical profile. Clin Exp Nephrol.

[8] Coutinho JM, Zuurbier SM, Stam J (2014). Declining mortality in cerebral venous thrombosis: a systematic review. Stroke.

[9] Deepyi S, Jasmina A, Akshay K. Thromboembolic complications in childhood nephrotic syndrome: a clinical profile.Clin Exp Nephrol,2014,18(5):803–813.doi: 10.1007/s10157-013-0917 -2. 10.1007/s10157-013-0917-2 24346593

[10] Kerlin BA, Ayoob R, Smoyer WE (2012). Epidemiology and pathophysiology of nephrotic syndrome-associated thromboembolic disease. Clin J Am Soc Nephrol.

[11] Cristoforo T, McKinley G, Ambrosio P (2021). Saddle pulmonary embolism in a pediatric patient with nephrotic syndrome and recent COVID-19 pneumonia: a case report. Am J Emerg Med.

[12] Hussein MH, Alabdaljabar MS, Alfagyh N, Badran M, Alamiri K. Splanchnic venous thrombosis in a nephrotic patient following COVID-19 infection: a case report.BMC Nephrol. 2021 Dec29;22(1):420. doi: 10.1186/s12882-021-02643-0. 10.1186/s12882-021-02643-0 PMC8715408 34965863

[13] Rostom A, Dube C, Cranney A, Rockville, MD). (: Agency for Healthcare Research and Quality (US);(Evidence Reports/Technology Assessments, No. 104.) Appendix D. Quality Assessment Forms. Celiac Disease. 2004. Sep[J].

[14] Xian-Tao Z, Hui L, Xi CH (2012).

[15] Carpenter SL, Goldman J, Sherman AK. Association of infections and venous thromboembolism in hospitalized children with nephrotic syndrome. Pediatr Nephrol. 2019 Feb;34(2):261–7. 10.1007/s00467-018-4072-6. 10.1007/s00467-018-4072-6 PMC6628263 30194664

[16] Tavil B, Kara F, Topaloglu R (2015). Case series of thromboembolic complications in childhood nephrotic syndrome: Hacettepe experience. Clin Exp Nephrol.

[17] Kerlin BA, Blatt NB, Fuh B (2009). Epidemiology and risk factors for thromboembolic complications of childhood nephrotic syndrome: a Midwest Pediatric Nephrology Consortium (MWPNC) study. J Pediatr.

[18] Mehls O, Andrassy K, Koderisch J (1987). Hemostasis and thromboembolism in children with nephrotic syndrome: differences from adults. J Pediatr.

[19] Lv YL, Guan N, Ding J, Yao Y (2020). Spectrum of thrombotic complications and their outcomes in chinese children with primary nephrotic syndrome. Ital J Pediatr.

[20] Lilova MI, Velkovski IG, Topalov IB (2000). Thromboembolic complications in children with nephrotic syndrome in Bulgaria (1974–1996). Pediatr Nephrol.

[21] Xian-Yan L, Xi-Qiang D (2016). Risk factors of primary nephrotic syndrome complicated with thrombosis in children. J Clin Pediatr.

[22] Hull RP, Goldsmith DJ (2008). Nephrotic syndrome in adults. BMJ.

[23] Mirrakhimov AE, Ali AM, Barbaryan A (2014). Primary nephrotic syndrome in adults as a risk factor for pulmonary embolism: an up-to-date review of the literature. Int J Nephrol.

[24] Scheres LJJ, Lijfering WM, Cannegieter SC (2018). Current and future burden of venous thrombosis: not simply predictable. Res Pract Thromb Haemost.

[25] Lachambre G, Proulle V, Bader-Meunier B. Misdiagnosis of venous thrombosis in childhood. Arch Pediatr. 2005 Feb;12(2):180-2. French. doi: 10.1016/j.arcped.2004.11.009. 10.1016/j.arcped.2004.11.009 15694545

[26] Leslom AN, Alrawiah ZMS, Al-Asmari AMA (2020). Prevalence of pulmonary thromboembolism in nephrotic syndrome patients: a systematic review and meta-analysis. J Family Med Prim Care.

[27] Nagalla VK, Raju SB, Ramesh Bura NR (2021). Arterial thrombosis Associated with factor V Leiden Mutation in a child with nephrotic syndrome. Indian J Nephrol.

[28] KDIGO Membranous nephropathy[J] (2021). Kidney Int.

[29] Monagle P, Chan AKC, Goldenberg NA. Antithrombotic therapy in neonates and children: Antithrombotic Therapy and Prevention of Thrombosis, 9th ed: American College of Chest Physicians Evidence-Based Clinical Practice Guidelines [published correction appears in Chest. 2014 Dec;146(6):1694. Dosage error in article text] [published correction appears in Chest. 2014 Nov;146(5):1422]. Chest. 2012;141(2 Suppl):e737S-e801S. doi:10.1378/chest.11-2308. 10.1378/chest.11-2308 PMC3278066 22315277

